# Exploring Consumer Perceptions and Economic Burden of Onchocerciasis on Households in Enugu State, South-East Nigeria

**DOI:** 10.1371/journal.pntd.0004231

**Published:** 2015-11-30

**Authors:** Ogochukwu Ibe, Obinna Onwujekwe, Benjamin Uzochukwu, Miriam Ajuba, Paul Okonkwo

**Affiliations:** 1 Health Policy Research Group, Department of Pharmacology and Therapeutics, College of Medicine, University of Nigeria, Enugu, Nigeria; 2 Department of Health Administration and Management, Faculty of Health Sciences, University of Nigeria, Enugu, Nigeria; 3 Department of Community Medicine, College of Medicine, University of Nigeria, Enugu, Nigeria; 4 Department of Community Medicine, Enugu State College of Science and Technology, Enugu, Nigeria; 5 Department of Pharmacology and Therapeutics, College of Medicine, University of Nigeria, Enugu, Nigeria; Santa Fe Institute, UNITED STATES

## Abstract

**Introduction:**

Onchocerciasis or river blindness constitutes a major burden to households especially in resource-poor settings, causing a significant reduction in household productivity. There has been renewed interest from policy makers to reduce the burden of Neglected Tropical Diseases (NTDs) such as onchocerciasis on individuals and households. This paper provides new information on the patient’s perceptions of onchocerciasis and its economic burden on households in South-eastern Nigeria. The information will be useful to health providers and policy makers for evidence-informed resource allocation decisions.

**Methods:**

Information was generated from a cross-sectional household survey conducted in Achi community, Oji River Local Government Area (LGA) of Enugu State, Southeast Nigeria. A pre-tested interviewer-administered questionnaire was used to collect data. A total of 747 households were visited randomly and data were collected using pre-tested interviewer administered questionnaire from 370 respondents. The respondents’ knowledge of the cause of symptoms of the disease, costs incurred for seeking treatment and productivity losses were elicited. Data were analyzed using tabulations and inferential statistics. A socio-economic status (SES) index was used to disaggregate some key variables by SES quintiles for equity analysis.

**Results:**

Many people had more than one type of manifestation of onchocerciasis. However, more than half of the respondents (57%) had no knowledge of the cause of their symptoms. Male respondents had significantly more knowledge of the cause of symptoms than females (P = 0.04) but knowledge did not differ across SES (P = 0.82). The average monthly treatment cost per respondent was US$ 14.0. Drug cost (US$10) made up about 72% of total treatment cost. The per capita productivity loss among patients was US$16 and it was higher in the poorest (Q1) (US$20) and the third SES quintiles (Q3) (US$21). The average monthly productivity loss among caregivers was US$3.5.

**Conclusion:**

Onchocerciasis still constitutes considerable economic burden on patients due to the high cost of treatment and productivity loss. Prioritizing domestic resource allocation for the treatment of onchocerciasis is important for significant and sustained reduction in the burden of the disease. In addition, focused health promotion interventions such as health education campaigns should be scaled up in onchocerciasis-endemic communities.

## Author Summary

Onchocerciasis is a public health problem in Nigeria, especially among the poor living in endemic communities. There is a dearth of evidence on the burden of onchocerciasis and studies suggest poor knowledge of the cause of onchocerciasis. This information could facilitate evidence-informed decisions on resource allocation towards the control of this neglected tropical disease. A cross-sectional survey was used to assess the knowledge of disease causation among patients, costs incurred for seeking treatment and productivity losses. About 43% had no knowledge of what caused their symptoms. The average monthly treatment cost per respondent was US$ 14.0. Drug cost (US$10) made up 72% of total treatment cost. The per capita productivity loss among patients was $16 and it was higher among the least poor (Q1) (US$20) and the poor SES (Q3) (US$21). The average cost of lost productivity among caregivers was US $3.5. These findings suggest that onchocerciasis still constitutes considerable economic burden on patients due to the high cost of treatment and lost productivity. This survey provided a measure of patient treatment cost in a setting with paucity of information. It also shows that targeted health education campaigns remain a fundamental policy option.

## Introduction

Onchocerciasis, otherwise called river blindness is one of the Neglected Tropical Diseases (NTDs) that constitute a public health problem [1]. It is commonly a burden to affected households and results to significant reduction in household productivity [2]. The disease is predominantly chronic with low mortality, and occurs largely in settings with low income, low disease awareness and little access to treatment [3]. These important characteristics of the disease contribute to its neglect as a priority public health problem.

Onchocerciasis is spread by the bites of small black flies (*Simulium species*) that breed along fast flowing rivers and streams, hence is more widespread in communities sited nearby. The characteristic symptoms of onchocerciasis often associated with long-term exposure to infection are particularly exasperating and disabling [4]. These symptomatic effects are both dermal and ocular and include atrophy of the skin (lizard skin), itching or pruritis, ocular lesion and nodules which are primarily caused by the presence of the microfilariae (the immature larval forms of the *Onchocerca volvulus)* in the subcutaneous tissue. People with heavy infections could have one or more of the three main conditions: dermatitis, eye lesions, and/or subcutaneous nodules [5]. The disease is reportedly the cause of 60% blindness in different parts of Africa, significantly affecting households socio-economic development [6], though levels of infection in the African Programme for Onchocerciasis (APOC) target areas are expected to fall drastically by 2015 [7].

The mainstay of the control of onchocerciasis is through the Community Directed Treatment with Ivermectin (CDTI) strategy [8]. This strategy involves delivering the treatment drugs for onchocerciasis to households via community volunteers [9]. The CDTI strategy is an initiative of the APOC, adopted in 1995 in 16 endemic countries, including Nigeria [9, 10] and have been in existence ever since. However, global attention and resources have shifted to more visible diseases such as malaria and HIV/AIDS, with less attention and resources devoted to the control of onchocerciasis and reduction of its economic burden. Recently, the World Health Organization (WHO) advised countries to aim towards the elimination of onchocerciasis by 2025 [11]. Achieving this mandate will require evidence-based resource priority setting towards more focused and sustainable programmatic approaches.

There is a dearth of empirical evidence on the economic burden of onchocerciasis on households in Nigeria, especially in terms of costs of treatment-seeking and lost productivity. Also, whilst the sociological and health effects of onchocerciasis have been documented, there is still paucity of information on the people’s knowledge and perceptions of the disease. In Tanzania, affected individuals were found unable to undertake their normal activities for an average duration of 3.7 days in a month [12]. In a multi-country study in Nigeria, Ethiopia and Sudan, onchocerciasis was responsible for poor school performance and a higher drop-out rate among infected children (due to itching, lack of sleep, visual impairment etc) [13]. Furthermore, low productivity, low income and higher healthcare related costs were found among infected adults [13]. Another study found that patients with ocular lesion reported giving up jobs because of their visual impairment, which led to loss of personal and household economic productivity in many cases [14].

Improved consumer knowledge of disease causation is considered a prerequisite for any disease control efforts. Better knowledge is shown to have a positive effect on prevention, treatment seeking and adherence to treatment, hence facilitates reductions in the economic burden of the disease [15]. Earlier studies report a low level of knowledge about the etiology of onchocercal symptoms which often led to inappropriate treatment seeking and overall disease management [16, 17]. Reports also suggest clear disconnect between symptoms of the disease and its cause for example, it has been reported that patients attributed their symptoms to the aging process or other blood-related conditions, while others perceived the various ocular and dermal symptoms of onchocerciasis as unrelated diseases, but rarely attributing the disease to black fly bites [14, 18].

This paper presents new information on households’ economic burden, including productivity losses due to onchocerciasis in Southeast Nigeria. It also explored patient’s knowledge of the cause and symptoms of onchocerciasis. The information is required for evidence-informed decisions on resource allocation towards the control of this neglected tropical disease.

## Methods

### Study design

The study was a cross-sectional household survey conducted in Achi community in Oji River Local Government Area (LGA) of Enugu State, Southeast Nigeria. The community was purposively chosen in consultation with the onchocerciasis Unit of the Enugu State Ministry of Health because the disease is endemic in this community. Achi is a rural community situated about 20 kilometres East of Enugu the capital of Enugu State. Subsistence farming provides employment for over 85% of the population in the community. Two villages were randomly selected from the community as the study sites.

### Sampling and sampling size

The study involved households with persons living with signs and symptoms of onchocerciasis. Simple random sampling was used to select households to be visited and screened for the presence of any individual with observable manifestations of the disease through physical examination of the skin. Those with observable signs of onchocerciasis manifestations were included in the study. Physical examinations were carried out by selected interviewers extensively trained for over 5 days by the study researchers and a local health worker on the different symptoms and aspects of the disease and how to administer the questionnaire. Most of the interviewers were from the locality and had previously been involved in other onchocerciasis-related community surveys in the area. Images of the different physical manifestations of onchocerciasis were used for the training and in addition, individuals who had different observable signs and symptoms of onchocerciasis, with their consent, were brought to the training, hence the interviewers were well conversant with the different symptoms.

A pre-tested interviewer-administered questionnaire was the data collection tool. The questionnaires were administered to those households that met the inclusion criteria. Questionnaires were pre-tested before the survey in a different LGA where the disease is also common. The pre-test was used to refine and modify the questionnaire to improve clarity of questions and context appropriateness.

The minimum sample size was calculated, using a power of 80%, 95% confidence level and a prevalence of 67% [19], which gave a sample size of 334. This was increased to 370 to control for non-responses. Households were visited in turn until the required sample size was reached.

#### Variables

The questionnaire has sections on demographic details of respondents, knowledge and perception of symptoms, cost of seeking treatment and productivity losses. A one month recall period was used in collection of cost data. Direct costs included those incurred on transport to and from treatment facility, consultation, drugs and food for the most recent treatment sought.

To evaluate time lost to illness, respondents were asked how many days in the past month that patient or caregiver missed doing their normal work due to their illness or taking care of a patient. For the purpose of this study a caregiver was defined as any household member who provided assistance and support towards the patient’s financial and physical needs as necessary. Respondents were encouraged to consult with other household members to enable accurate recollection of time lost.

#### Data analysis

Tabulations and inferential statistics were the data analytical tools. Lost productivity was quantified, using the Human Capital Approach (HCA) which expresses time loss as the product of missed workdays and wage per day [20]. Wage per day was calculated from the prevailing national minimum wage of fifteen thousand naira (15000) per month at the time of the study.

In order to examine inequities in some of the key variables, a socio-economic status (SES) index was created, using Principal Components Analysis (PCA) [21]. The inputs to the PCA were the information on ownership of key household assets which included motorcar, motorcycle, radio, refrigerator, television set, electric iron, kerosene lamp and bicycle. The SES index was used to divide the households into quintiles to explore the disaggregation of the key variables by SES. The SES distribution of respondents were Q1 (Most poor), Q2 (Second), Q3 (Third), Q4 (Fourth), and Q5 (Richest). Test of significance was set at 5% (p value <0.05).

### Ethics

Ethical approval was obtained for the study from the University of Nigeria Ethics Review Board Enugu. Written informed consent was obtained from each respondent before administering the questionnaire.

## Results

A total of 747 households were visited and data were collected, using pre-tested interviewer-administered questionnaire from 370 respondents who met the eligibility criteria. Analysis was based on 361 questionnaires with the exclusion of 7 with incomplete information. The characteristics of respondents are shown in Table 1.

**Table 1:** Socio-demographic characteristics of respondents.

Variable		N = 361n (%)
Age	Mean(SD)	46 (15.1)
**Gender**	Female	234 (64.8)
	Male	127 (35.2)
**Highest level of education** ^**i**^	Primary	132 (36.5)
	Secondary	61 (16.8)
	Tertiary	19 (5.2)
	Not educated	142 (39.2)
	Others	3 (0.8)
**Years spent in school**	Mean (SD)	4.7 (4.8)
**Marital status**	Monogamously married	207 (57.1)
	Polygamously married	16 (4.4)
	Divorced	3 (1.0)
	Widowed	73 (20.2)
	Unmarried	57 (15.7)
	Refused to answer	3 (1.0)
	Other	1 (0.2)
**Main source of income** ^**ii**^	Farming	162 (45.0)
	Petty trading	32 (9.0)
	Self employed	69 (20.0)
	Daily paid labourer	5 (1.0)
	Employed in the public sector	17 (5.0)
	Employed in private sector	5 (1.0)
	Unemployed	63 (17.0)
	Other	9 (2.0)
**Distribution of respondents by SES**	Q1 (Poorest)	73 (20.3)
	Q2 (Second)	72 (20.0)
	Q3 (Third)	73 (20.3)
	Q4 (Fourth)	72 (20.0)
	Q5 (Richest)	70 (19.4)
**No of household residents**	Mean (SD)	4 (0.1)

i Missing 4 responses

ii missing 15 responses

iii missing one response

### Socio-demographic characteristics of respondents from the questionnaire interview

Table 1 show that most of the respondents were middle-aged (46yrs) and almost two-thirds were females (65%). More than a third were not educated (39%). The educated ones spent an average of 5 years in school and the highest level of education attained by many respondents was primary school. Most of the respondents were monogamously married (57%) and almost half of the respondents were farmers (45%). The average number of household residents was four (4).

### Types of symptoms and patients’ knowledge of manifestations

Almost half (45.4%) of the respondents had more than one type of symptom but the commonest manifestation was palpable nodule (86.9%), followed by lizard skin (4.4%), itching (12.7%) and ocular lesion (12.5%) (Table 2). Less than half of the respondents (42.6%) had knowledge of the cause of their symptoms. Male respondents had significantly better knowledge than females (P = 0.04) but knowledge did not differ across SES (P = 0.82).

**Table 2:** Types of symptoms experienced and patient’s knowledge of manifestations.

Variable		N = 361n (%)
**Types of symptoms experienced**	Swollen limb	7 (1.9)
	Craw craw/itching	64 (17.7)
	Spotty pigmentation	43 (11.9)
	Lizard skin	16 (4.4)
	Palpable nodule	314 (86.9)
	Hanging groin	9 (2.4)
	Blindness	45 (12.5)
	Others	1 (0.3)
**Patients with more than one symptom**	Yes	164 (45.4)
	Male	62 (37.8)
	Female	102 (62.2)
**Average number of symptoms**	Mean (SD)	1.60 (0.79)
**Do you know the cause of your symptom(s)?**	Yes	154 (42.6)
	Male	67 (52.7)
	Female	87 (37.2)
	Chi^2^ (P-value)	**8.16 (0.04)**
**Knowledge of symptoms by SES**	Q1 (Poorest)	28 (38.7)
	Q2 (Second)	31 (43.0)
	Q3 (Third)	33 (45.2)
	Q4 (Fourth)	29 (40.3)
	Q5 (Richest)	33 (47.1)
	Chi^2^ (P-value)	**1.48 (0.82)**

### Patterns of treatment seeking for patients manifestations

Overall, only about 14% of respondents sought treatment in the one month preceding the survey, 16% in the one year preceding survey and more than half (60%) had never sought any treatment for their condition (Table 3). The table also shows that about half (54.2%) of those that sought treatment did so from a private facility.

**Table 3:** Patients treatment seeking for manifestations.

Variable		N = 361n (%)
Did you seek treatment in past month?*	Yes	48 (13.5)
	No	307 (86.5)
**Last time treatment was sought**	Past month	48 (13.5)
	< 6 months	17 (4.7)
	> 6 months	11 (3.0)
	> 6 months	70 (19.4)
	Never sought treatment	209 (57.9)
**Place where treatment was sought**	Public facility	6 (12.5)
	Private facility	26 (54.2)
	Traditional	10 (20.8)
	Other	6 (12.5)

### Treatment costs for an out-patient visit

Out of the 48 respondents that reported having sought treatment, the average monthly treatment cost per out-patient visit was US$ 14.0 with drug cost (US$ 10) contributing about 72% of total cost (Table 4). The average transport cost was US$ 0.9, representing about 6.4% of total cost.

**Table 4:** Treatment costs for an out-patient visit.

Cost categoryN = 48	Naira	US Dollar
	Mean (SD)	Mean (SD)
**Transport cost N = 45**	148.8 (218.3)	0.9 (1.4)
**Consultation N = 41**	68.7 (221.7)	0.4 (1.5)
**Drug N = 44**	1580.9 (1968.6)	10.0 (13.1)
**Food N = 40**	159.0 (488.0)	1.1 (3.2)
**Total out-patient cost**	2107.8 (2964.3)	14.0 (19.7)

### Average monthly SES differences in the number of work days lost due to illness

Overall, about 28% patients reported they have had to miss work in the one month prior to the day of interview due to their onchocerciasis-related illness for an average of 3 days (Table 5). Patients in the poorest (Q1) and the third (Q3) quintile groups missed work for greater number of days (4 days) than the rest of the SES groups. However, fewer number of caregivers had entirely missed work and for an average of 0.7 days. Overall, the illness limited an average of 8 days of work for the patients. Patients in the poorest quintile lost more productive days on average (9 days) compared to the richest who lost 7 days in a month. There were no statistically significant differences in the results (Table 5).

**Table 5:** Average monthly SES differences in the number of work days lost due to illness.

Variable	Q1 N = 73	Q2 N = 72	Q3 N = 73	Q4 N = 72	Q5 N = 70	Chi2 (p-value)	Combined N = 361
Patient lost an entire day of work?*Yes	23 (31.5)	22 (30.6)	14 (19.2)	19 (26.4)	21 (30.0)	3.72 (0.44)	99 (27.5)
**Number of days Mean (SD)**	4.0 (8.3)	2.9 (6.8)	4.2 (10.1)	2.8 (7.1)	2.1 (5.1)	0.89 (0.92)	3.2 (7.7)
**Caregiver lost an entire day of work?Yes**	8.0 (11.0)	3.0 (4.2)	2.0 (2.7)	3.0 (4.2)	10.0 (14.3)	10.9 (0.03)	26.0 (7.2)
Number of days **Mean (SD)	1.7 (5.64)	0.5 (2.2)	0.3 (2.1)	0.2 (1.40)	1.0 (3.3)	2.2 (0.70)	0.7 (3.3)
**Work limitation for patients**	43 (58.9)	40 (55.6)	40 (54.8)	35 (48.6)	38 (55.1)	1.62 (0.80)	196 (54.6)
**Number of days Mean (SD)**	9.6 (12.1)	7.6 (10.8)	7.9 (11.1)	6.7 (10.1)	6.7 (10.3)	1.44 (0.83)	7.7 (10.9)

*missing 1 response

** missing 3 responses

### Average cost of monthly productivity loss by SES

The average cost of patient’s productivity loss was $16.0 and it was higher among patients in the poorest (US$20) and the third quintiles (US$21). Similarly, the cost of productivity loss was also highest among caregivers in the poorest quintiles (US$8.5) compared to those in the richest quintile (US$5). Overall, the total cost of lost productivity was US$19.5 (Table 6).

**Table 6:** Average monthly cost of productivity loss by SES.

Variable	Q1 N = 73	Q2 N = 72	Q3 N = 73	Q4 N = 72	Q5 N = 70	Combined N = 361
**Cost of patient’s missed work days (In naira)**	3000	2175	3150	2100	1575	2400
**Cost of patient’s missed work days (In US$)**	20	14.5	21	14	10.5	16
**Cost of caregiver’s missed work days (In naira)**	1275	375	225	150	750	525
**Cost of caregiver’s missed work days (In US$)**	8.5	2.5	1.5	1	5	3.5

## Discussion

The study assessed the economic burden of onchocerciasis illness and patient’s knowledge of the disease in Southeast Nigeria. The findings show that patients incur considerable economic burden from onchocerciasis from treatment costs and lost productivity. Since treatment was not usually sought for many symptomatic conditions, the brunt of the burden arises from the indirect economic loss represented by loss of productive time of the sufferers and their caregivers.

Evaluations of the economic burden of onchocerciasis have often focused on costs to the provider [22] [23] with little attention paid to the potential cost of treatments to patients. There has also been limited empirical estimation of the productivity losses to patients, especially in endemic communities in Nigeria. Earlier studies have shown the negative impact of onchocerciasis on productivity of farmers [2, 24, 25] For example, sufferers have been found to have spent additional $8.10 and 6.75 hours over a period in seeking care [26]. The treatment cost of US$14 reported in this study is an indication that those who seek treatment for onchocerciasis manifestations incur significant cost, which may constitute an important component of the socio-economic burden of the disease in endemic communities.

The reported average daily work loss (represented by total work absence) found in this study is less than those reported in earlier studies [27], However, the substantial work-limited-days raises an issue of concern. A study from western Nigeria found that majority of the patients (60%) lost 14 days monthly and others (37.8%) lost between 7 and 14 days, respectively, due to illnesses resulting from onchocerciasis [27]. Another study showed that onchocerciasis decreased the daily wage of workers by approximately 16%, those with intermediate manifestations of onchocercial skin diseases were found to earn approximately 10% lower than non-sufferers [28]. Findings from a study in Nigeria reported that farmers with onchocercial skin disease (OSD) had an overall lower standard of living, as indicated by their ownership of fewer personal wealth indicators such as motorcycles, radios, iron roofing and cement-plastered houses [29]. Other researchers argue that in blackfly-infested communities, there were generally low levels of productivity by farmers [2, 19]. These losses are often due to the fact that the ocular and dermal symptoms of onchocerciasis make it difficult for people to work optimally, thus diminishing their output and income generating capacity [30].

The average monthly cost of productivity loss of US$19.5 from patients and caregivers could be considered a huge economic burden on the affected individuals and their families, largely subsistence farmers whose consumption depends largely on their productive ability. Our findings also raises some equity concerns in both productivity losses and associated costs, given that lost productivity was greater among the poorest socio-economic. The absence of risk-pooling mechanisms in Nigeria to protect households from financial shocks that could arise from common healthcare expenditures mean that households and more importantly, poorer households are likely to experience a disproportionate burden of the disease.

Estimating the level of productivity loss due to onchocerciasis often presents a peculiar challenge as affected individuals are the poor people less likely in paid employment [31]. There may be limitations in using the national minimum wage to estimate productivity loss because it may have overestimated or underrated the burden of the disease, given that the wage per day may not accurately reflect respondent’s earnings, hence the findings of this study should be interpreted with caution. But using this approach allows a uniform way of evaluating lost productivity, given the multiplicity of job types and earnings in rural communities.

The proportion of respondents with ocular lesion and lizard skin compares to the reported finding of 7.5% of lizard skin and 14% eye lesion by another study in Nigeria[14]. However, an earlier study in Achi, Oji River shows that a majority (44.3%) of subjects had nodules, 18% had lizard skin and 20% had leopard skin [19]. The proportion of subjects with nodules was less than found in our study. However, our study shows reductions in the appearance of other skin and ocular conditions which could be an indication of gains in the widespread distribution of ivermectin drug. In the western part of Nigeria, leopard skin was reportedly the commonest manifestation experienced by respondents and the proportions of patients with nodules were also much less (10%) when compared to our study [14]. This could be explained by the geographic locale of the studies and possible differences in transmission patterns of onchocerciasis [7]. Elsewhere, nodules were considered most common and least worrisome feature of the disease [32].

The fact that just about half of the patients had knowledge of the cause of their symptoms shows that there is still a huge gap in knowledge about the cause of onchocerciasis even in endemic areas. Poor knowledge was found in another study in Southwest Nigeria, where study subjects failed to establish the link between the vector and the symptoms of the disease [18]. Other studies in Nigeria and elsewhere have shown similar findings [33, 34]. Although we did not find any socio-economic differences in knowledge of cause of the disease, females generally had poor knowledge of cause of manifestation even though more women had the disease. This could be that women are less exposed to health messages and awareness campaigns and poor knowledge could result in neglect of protection and early treatment seeking which may exacerbate disease morbidity [35].

A key limitation, of this study is the sole reliance on physical examination of symptoms by non-medical persons which may have lowered the precision of diagnosis. Future costing studies could combine physical examination and precise clinical methods, including biopsies and tests for visual acuity [36]. However, careful training and supervision of examiners used in this study may well have reduced this limitation to a barest minimum. A further limitation is that the sample may not be representative of the entire communities in Southeast Nigeria since it was done in endemic community. It may be necessary in the future, compare findings from highly, low and non-endemic communities. More work is also necessary to strengthen the evidence base of the economic burden for improved resource allocation decisions. An area for future study could also be to conduct large-scale longitudinal studies examining the burden of disease overtime, the level of income depletion and its effects on households of different socioeconomic status.

In conclusion, this study has provided important but scarce information on the patient level cost of treatment seeking and productivity due to onchocerciasis. It shows that onchocerciasis still constitutes considerable burden to patients due to the cost of losses in productivity and treatment seeking. This burden is certain to have negative impact on affected household subsistence. Concerted efforts are required to accelerate actions towards the control of onchocerciasis so as to reduce its economic burden. Prioritizing domestic resource allocation for the treatment of onchocerciasis, especially strengthening, scaling-up and sustaining the CDTI strategy over the long run is key to significant and sustained reduction in the burden of onchocerciasis. This will require a strong political will. In addition, health promotion interventions such as health education campaigns should be scaled up in onchocerciasis-endemic communities.

## Supporting Information

S1 Text Questionnaire. (DOCX)
