# Supplementary material for: Exploring Consumer Perceptions and Economic Burden of Onchocerciasis on Households in Enugu State, South-East Nigeria
Source: PLoS Negl Trop Dis. 2015 Nov 30;9(11):e0004231. doi: 10.1371/journal.pntd.0004231 (PMC4664248; doi:10.1371/journal.pntd.0004231)
Supplement: S1 Text — (DOCX) [file pntd.0004231.s001.docx]

**ASSESSING THE SOCIO-ECONOMIC IMPACT OF ONCHOCERCIASIS IN OJI RIVER ENUGU STATE**

**THE QUESTIONNAIRE**

**SECTION A: BACKGROUND INFORMATION**

**(*Instruction to interviewer:*** *This part should be filled by the interviewer before the start of the interview. The first respondent should be coded 01, the second 02 etc. At any point in time the respondent can consult with other household members to answer the questions if they need to)*

1. **Interviewer name ……………………………….. ………… *Site code* *personal code***
2. **Interviewer code. ………………/…………………………**

***Site code interviewer code personal code***

1. **Respondent code ……………/……………………./…………**
2. **Name of study site …………………………………………...**
3. **Code of study site …………………………………………..**
4. **Time of interview ………………/………………………….**
5. **Date of interview *DD……….MM……….YR*…………………….**

**OBTAINING CONSENT**

**(*Instruction to interviewer:*** *Please refer to SOP for obtaining consent****.*** *Prior to the start of the interview explain the study to the respondent give him/her the information sheet so they can read through. Also explain that they need to sign a consent form. Ensure that all boxes are properly filled, if the answer is yes write 1 in the box and if no write 0 unless otherwise stated*)

**8.** Has the respondent given consent to complete the questionnaire

1=yes [ ] go to 10

0= No [ ] go to 9

9. If they refused what was the reason given

a. too busy [ ]

b. not interested [ ]

c. refuse to answer [ ]

d. other (describe) [ ]

**SCREENING**

*This study is an assessment of the socio-economic impact of Onchocerciasis (Isi anyaocha) on household in this community. Before we proceed with getting some information from you, we would like to know if this household is eligible to participate. To determine this we would need to physically examine members of your household as we have explained to you earlier. The following information you’ll give us will help us to determine your eligibility. Refer to SOP for screening.*

| 10. Do you or any member of your household experience any of the following manifestations? (Interviewer to also critically observe) 1=Yes, 0 = No   1. Swelling of limb [ ] 2. Craw craw/ intense itching (oko nyimbo) [ ] 3. Spotty pigmentation on skin (okpa ocha) [ ] 4. Hanging groin [ ] 5. Ageing/lizard skin (wrinkled with shiny fragile appearance, scarring and loss of elasticity) [ ] 6. Palpable noodles (Akpu) [ ] 7. Blindness (isi anyaocha) [ ] |
| --- |

***For eligible households***

11a. Do you know the cause of the manifestation you experienced/have [ ]

If yes go to 11b. if No go to 12. (1=Yes, 0 = No)

11 b. What do you think is the cause ………………………………

**SECTION B: *General socio-economic and demographic characteristics***

*(Interviewer to read to respondent: I want to now ask you questions concerning your household; this will take only a little while***)**

1. What is your age [ ]
2. Gender of respondent (*Interviewer to record*) [ ]

Male = 1

Female = 0

1. What is your status in this household [ ]

1= Household head 0 = representative of household head

1. What was your highest level of education? 1= yes 0 = no
2. Primary [ ]
3. Secondary [ ]
4. Tertiary [ ]
5. Not educated [ ]
6. Other (describe) [ ]
7. Refuses to answer[ ]
8. Don’t know [ ]
9. What was the total number of years you spent in school? [ ]
10. What is your current marital status? 1= Yes 0 = No
    1. Married with one wife/husband [ ]
    2. Married with many wives [ ]
    3. Divorced/Separated [ ]
    4. Widowed [ ]
    5. unmarried [ ]
    6. Refuse to answer [ ]
    7. Don’t know [ ]
    8. Other (describe) …………………………..
11. What is your **main** source of income? 1= Yes 0 =No
12. Farming [ ]
13. Petty trading [ ]
14. Self employed [ ]
15. Daily paid laborer [ ]
16. Employed in the public sector [ ]
17. Employed in the private sector [ ]
18. Unemployed [ ]
19. Other (describe) [ ] ………………………….
20. In addition to yourself, how many people live in this household [ ]

**SECTION C*: treatment seeking and costs of seeking treatment***

***(****I would want to ask you about where you sought treatment and the costs you have incurred in the* ***past month*** *due to the illness****. Instruction to interviewer:*** *the respondent can consult with other members of the household to answer the question if need be)*

8. Have sought treatment for this condition **in the past month** [ ] 1 = Yes 0= No

(*If* ***YES go to 16****, if* ***NO go to 15***)

9. When was the last time you sought treatment? 1 = Yes 0 = No

a**.** More than one month [ ]

b. Two months ago [ ]

c. More than 2 months [ ]

d. More than 6 months [ ]

e. Up to 1 year [ ]

f. More than one year [ ]

g. Never sought treatment [ ]

h. Other (describe) [ ]

***Note: If patient has never sought treatment, go to section D***

10. Where did you seek treatment? 1= Yes 0 = No

a. public hospital [ ]

b. private hospital [ ]

c. missionary hospital [ ]

d. primary health centre [ ]

e. patent medicine dealer[ ]

f. traditional healer [ ]

g. self treatment [ ]

h. other (describe) [ ]

11. How did you travel to the facility? 1 = Yes 0 =No

1. Private car [ ]
2. Public taxi/bus [ ]
3. Motorcycle [ ]
4. Bicycle [ ]
5. Walk [ ]
6. Other describe [ ]

**Please can you tell me about the cost you incurred in your last out-patient visit in past month?** *Interviewer record where the cost applies.*

| **Category of cost** | **Amount in Naira** |
| --- | --- |
| 12. Health Card / Registration | [_________] Naira |
| 13. Consultation Fee | [_________] Naira |
| 14. Test | [__________] Naira |
| 15. Cost of drugs | [__________] Naira |
| 16. Cost of any food (if taken as a result of treatment) | [_________] Naira |
| 17. Other Costs (1) specify  [______________________________________] | [_________] Naira |
| 18. Other Costs (2) specify  [_______________________________________] | [_________] Naira |
| 19. Total (*interviewer add up)* | [__________] Naira |

20. Did you visit the facility alone or with a minder/caregiver? [ ] 1 = Yes 0 = No (if No, go to 22)

21. How much did the caregiver cost you? [ ] Naira

*(Total money spent on transportation, food and any other money spent on the caregiver during the hospital visit)*

22. Were you admitted at the facility? [ ] 1 = Yes 0 = No (***if No go to Section D***)

23. How many days were you admitted? [ ] days

**Please can you tell me about the cost you incurred in your last in-patient visit in past month?** *Interviewer record where the cost applies.*

| **Category of cost** | **Amount in Naira** |
| --- | --- |
| 24. Health Card / Registration | [_________] Naira |
| 25. Consultation Fee | [_________] Naira |
| 26. Test | [__________] Naira |
| 27. Cost of drugs | [__________] Naira |
| 28. Cost of any food (if taken as a result of treatment) | [_________] Naira |
| 29. Other Costs (1)  [_______________________________________________] | [_________] Naira |
| 30. Other Costs (2)  [_______________________________________________] | [_________] Naira |
| 31. Total (*interviewer add up)* | [__________] Naira |

32. Did a caregiver you visit the facility alone or with a minder/caregiver? [ ] 1 = Yes 0 = No (if No, go to section D)

33. How much did the caregiver cost you? [ ] Naira

*(****total money spent on transportation, food and any other money spent on the caregiver during the hospital visit)***

***SECTION D****:* ***Productivity loses***

***(Instruction to interviewer****: the respondent can consult with relevant household members to answer this section****)***

33. **In the past one month**, has the **sick person** missed an **entire day** of work because of this condition? [ ]

1. = Yes 0= No

34. Can you tell me the total number of days he/she missed work in the past month? [ ] days

35. **In the past one month**, has the **caregiver/minder** missed an **entire day** of work as a result of the patient’s sickness? [ ] 1 = Yes 0 = No

36. Can you tell me the total number of days in the past one month? [ ] Days

37. In the past one month has the sickness limited the amount of work that the patient is able to do [ ] yes =1, No = 0

38. Can you tell me the number of days in the past one month? [ ] days

***SECTION E: Household Expenditures on food items***

39. Can you tell me how much you spent in the **last one week** in buying the following food items *(****interviewer to read list and record the corresponding amount****)*

| **Items** | **What quantity** | **How much** | **Who bought** | **How many times in a year do your household buy this item (Period codes: 1 = weekly, 2 = monthly, 3 = every 3 months, 4 = twice a year, 5 = yearly)** |
| --- | --- | --- | --- | --- |
| Rice |  |  |  |  |
| Beans |  |  |  |  |
| Garri |  |  |  |  |
| Yam |  |  |  |  |
| Cocoyam |  |  |  |  |
| Fish |  |  |  |  |
| Meat |  |  |  |  |
| Others (name) |  |  |  |  |
| **Total** |  |  |  |  |

40**.** If the food items consumed in the last one week were produced by the household, how much would it be worth in the market if you were to sell them**.**

| **Item** | **Quantity** | **Amount** | **Total (Qty X Amt)** |
| --- | --- | --- | --- |
| Rice |  |  |  |
| Beans |  |  |  |
| Garri |  |  |  |
| Yam |  |  |  |
| Cocoyam |  |  |  |
| Fish |  |  |  |
| Meat |  |  |  |
| Others (name) |  |  |  |
| **Total** |  |  |  |

41. Total food cost: interviewer to add **39 + 40** [ ] Naira

***Expenditures on non-food items***

42. How often does your **household** spend money on the following items and how much do they spend.

| **Item** | **Period codes**  Weekly = 1, monthly = 2, quarterly = 3,  bi-annually = 4,  Annually **=** 5 | **Amount Spent** | **Annual expenditure**  **(interviewer to add up)** |
| --- | --- | --- | --- |
| a. Clothing |  |  |  |
| b. Rent |  |  |  |
| c. Household furniture, |  |  |  |
| d. Cooking fuel |  |  |  |
| e. Healthcare |  |  |  |
| f. Educational expenses |  |  |  |
| g. Other expenses (describe) |  |  |  |
| h. **TOTAL** |  |  |  |

**SECTION F**: ***Payment and disease coping mechanisms***

43. How did you pay for the **last treatment** received? (I will read out some payment options and please answer Yes or No) 1= Yes 0 = No

a. Paid in cash but was reimbursed by employer [ ]

b. Out-of pocket [ ]

c. Health insurance [ ]

d. Instalment [ ]

e. In-kind [ ]

f. others (describe) [ ]

44. How did you cope with payment for this illness (interviewer to read list) 1 = Yes 0 = No

a. used own money to pay [ ]

b. borrowed money [ ]

c. sold household movable asset [ ]

d. sold family land [ ]

e. took loan from bank/money lenders [ ]

f. community solidarity [ ]

g. was exempted from payment [ ]

h. someone else paid [ ]

i. others (describe)

45. In general how do you cope with all the costs due to this illness

(***Table summarizing household coping mechanism for the illness in the past one month)***

|  |
| --- |
|  |
|  |
|  |
|  |
|  |
|  |

**SECTION G***:* ***Household characteristics and asset holding***

(*Interviewer to read list*)

43. Can you tell me if any member of the household own any of the following items

1= Yes 0 = No

1. Motorcycle [ ]
2. Motor car [ ]
3. Bicycle [ ]

44. Does the household have a working/Functional

1. Electricity [ ]
2. Radio [ ]
3. Television [ ]
4. Fixed line/landline [ ]
5. Electric cooker [ ]
6. Iron [ ]
7. Kerosene lamp [ ]
8. Refrigerator [ ]

45. Including the respondent how many people live in this household? [ ]

46. How many rooms are there in total in this house? [ ]

47. How many are used for sleeping? [ ]

48. What is the main source of drinking water? 1 = Yes 0 = No

1. Private pipe [ ]
2. Public pipe [ ]
3. Well [ ]
4. Surface water/stream [ ]
5. Water tanker supplies [ ]
6. Rainwater [ ]
7. Other (describe) [ ]

49. What kind of toilet facility do members of the household have? 1 = Yes 0 = No

1. Private Pit toilet [ ]
2. Shared pit [ ]
3. Bush [ ]
4. Water closet [ ]
5. Other (describe) [ ]

50.Are you the owner of where you live or a tenant? [ ] 1 = owner 0 = tenant

51.What is the floor of your house made of? 1 = Yes 0 = No

1. Earth/sand [ ]
2. Wood [ ]
3. Cement [ ]
4. Carpet [ ]
5. Ceramic tiles [ ]
6. Don’t know [ ]
7. Other (describe) [ ]

THANK YOU.

Enumerator to record time that interview ended ……………..
